# Combined Cytolytic Effects of a Vaccinia Virus Encoding a Single Chain Trimer of MHC-I with a Tax-Epitope and Tax-Specific CTLs on HTLV-I-Infected Cells in a Rat Model

**DOI:** 10.1155/2014/902478

**Published:** 2014-03-27

**Authors:** Takashi Ohashi, Takafumi Nakamura, Minoru Kidokoro, Xianfeng Zhang, Hisatoshi Shida

**Affiliations:** ^1^Division of Molecular Virology, Institute for Genetic Medicine, Hokkaido University, Kita 15, Nishi 7, Kita-ku, Sapporo, Hokkaido 060-0815, Japan; ^2^Division of Integrative Bioscience, Department of Biomedical Science, Institute of Regenerative Medicine and Biofunction, Graduate School of Medical Science, Tottori University, Yonago, Tottori 683-8503, Japan; ^3^Department of Virology III, National Institute of Infectious Diseases, Musashimurayama, Tokyo 208-0011, Japan

## Abstract

Adult T cell leukemia (ATL) is a malignant lymphoproliferative disease caused by human T cell leukemia virus type I (HTLV-I). To develop an effective therapy against the disease, we have examined the oncolytic ability of an attenuated vaccinia virus (VV), LC16m8Δ (m8Δ), and an HTLV-I Tax-specific cytotoxic T lymphocyte (CTL) line, 4O1/C8, against an HTLV-I-infected rat T cell line, FPM1. Our results demonstrated that m8Δ was able to replicate in and lyse tumorigenic FPM1 cells but was incompetent to injure 4O1/C8 cells, suggesting the preferential cytolytic activity toward tumor cells. To further enhance the cytolysis of HTLV-I-infected cells, we modified m8Δ and obtained m8Δ/RT1AlSCTax180L, which can express a single chain trimer (SCT) of rat major histocompatibility complex class I with a Tax-epitope. Combined treatment with m8Δ/RT1AlSCTax180L and 4O1/C8 increased the cytolysis of FPM1V.EFGFP/8R cells, a CTL-resistant subclone of FPM1, compared with that using 4O1/C8 and m8Δ presenting an unrelated peptide, suggesting that the activation of 4O1/C8 by m8Δ/RT1AlSCTax180L further enhanced the killing of the tumorigenic HTLV-I-infected cells. Our results indicate that combined therapy of oncolytic VVs with SCTs and HTLV-I-specific CTLs may be effective for eradication of HTLV-I-infected cells, which evade from CTL lysis and potentially develop ATL.

## 1. Introduction

Human T cell leukemia virus type I (HTLV-I) is etiologically linked to adult T cell leukemia (ATL) [1, 2] and a chronic progressive neurological disorder termed HTLV-I-associated myelopathy/tropical spastic paraparesis (HAM/TSP) [3, 4].HTLV-I genome contains a unique 3′ region, designated as pX, which encodes the viral transactivator protein, Tax [5]. It is speculated that Tax plays a central role in HTLV-I associated immortalization and transformation of T cells, which may lead to the development of ATL [6]. In addition, Tax is also known as a major target protein recognized by cytotoxic T lymphocyte (CTL) of HTLV-I carriers [7]. A number of studies have reported that CTL responses were activated in HAM/TSP patients but were weak in ATL patients, suggesting that the T cell response could be one of the important determinants of the disease manifestation [8]. Since HTLV-I Tax-specific CTL can recognize and lyse ATL cells in vitro [9], it is conceivable that the low CTL activity in ATL patients is disadvantageous as it may allow uncontrolled proliferation and evolution of HTLV-I-infected cells in vivo. Indeed, Hasegawa et al. have reported that oral HTLV-I-infection induced HTLV-I-specific T cell tolerance and caused an elevation of the proviral loads and that reimmunization resulted in the recovery of the virus-specific T cell responses and the decrease of the proviral loads in a rat model system [10]. In addition, the development of ATL has been reported in HTLV-I carriers who received immunosuppressants during organ transplantation [11]. Increase of Tax-specific CTLs observed in ATL patients treated successfully with allogeneic hematopoietic stem cell transplantation (allo-HSCT) also suggests the importance of virus-specific CTLs to control the disease [12]. Thus, immune therapies to activate HTLV-I-specific CTLs are considered as novel attempts for the treatment of ATL. In this regard, we have previously demonstrated the therapeutic effect of Tax-coding DNA or peptide in a rat model of ATL-like disease [13, 14]. In addition, it has been recently reported that autologous Tax-specific CTLs showed therapeutic benefits in an animal model using NOG mice bearing primary ATL cells, suggesting the possible translation into a clinical use [15].

To improve therapeutic effects of immune therapy, it is important to consider tumor microenvironment, because tumor cells often induce a microenvironment, which favors the development of immunosuppressive populations of immune cells, such as myeloid-derived suppressor cells and regulatory T cells [16]. In HTLV-I carriers and ATL patients, various kinds of immunosuppressive events have been reported, indicating the importance of developing new strategies to eliminate HTLV-I-infected cells in such immunosuppressive environments [8]. One of powerful strategies to lyse tumor cells in an immunosuppressive microenvironment would be the use of replication-competent oncolytic viruses, because oncolytic virotherapy has been known to induce both direct tumor killing and local proinflammatory environments that help to reverse the immunosuppressive environment of tumors [17, 18]. As for HTLV-I infection, vesicular stomatitis virus (VSV) has been reported to have oncolytic activity against primary ATL cells [19]. Vaccinia virus (VV) has been also shown to be a good candidate for oncolytic virotherapies [20]. It has been already assessed in clinical trials and shown to selectively infect, replicate, and express transgene products in cancer tissues without damaging normal tissues [21]. We have previously constructed a highly attenuated VV, LC16m8Δ (m8Δ), which is genetically more stable than LC16m8 (m8), a naturally occurring counterpart of m8Δ, and less pathogenic than its parental LC16mO (mO) due to the deletion of B5R gene [22]. The safety of m8Δ has been already confirmed in clinical use of its natural counterpart; m8 has been safely administered to approximately 100,000 infants and 3,000 adults for smallpox vaccination and induced levels of immunity similar to those of the original Lister strain without serious side effects [23]. Moreover, Hikichi et al. have recently reported the oncolytic potential of m8Δ with regulated expression of B5R [24]. Thus, the application of m8Δ for the elimination of HTLV-I-infected cells should be possible.

It is well known that HTLV-I viral protein expression is suppressed in infected cells in the peripheral blood of the virus-infected individuals, probably due to either unidentified suppression mechanisms of HTLV-I expression or genetic and epigenetic changes in the viral genome [8]. This reduction of viral protein expression may cause the decrease of anti-HTLV-I immune responses. Downregulation of major histocompatibility complex class I (MHC-I) could also lead to the evasion of HTLV-I-infected cells from the virus-specific CTLs [25]. Thus, strategies to overcome the repression of viral antigen presentation in HTLV-I infected individuals should be also required to establish effective anti-HTLV-I therapies. Improving the ability to present antigen to proper CTLs could be one possible way to overcome the problem. Single chain trimers (SCTs) of MHC-I have been reported to possess the strong potential to stimulate antigen-specific T cells [26, 27]. In this system, all three components of MHC-I complexes, such as an antigen peptide, *β*_2_-microgrobulin (*β*_2_m), and MHC-I heavy chain, were covalently attached with flexible linkers. By connecting together the three components into a single chain chimeric protein, a complicated cellular machinery of antigen processing can be bypassed, leading to stable cell surface expression of MHC-I coupled with an antigenic peptide of interest. It has been recently reported that SCT-expressing DNA vaccine is able to break immune tolerance against self-antigen from melanoma, further supporting the potential of SCTs to clinical applications [28]. By applying SCT system to a rat model of HTLV-I infection, we have previously developed an activation and detection system of Tax-specific rat T cells and showed that SCTs with a Tax-epitope specifically recognize and activate Tax-specific CTLs [29]. In this study, to further improve the efficacy of CTL activation by SCTs, m8Δ was selected as a vector to express SCTs on the surface of HTLV-I-infected cells. Introduction of SCT coding sequence into the genome of m8Δ could generate novel therapeutic VVs, which possess abilities to both lyse tumor cells and activate tumor-specific CTLs. We further examined the combination effects of Tax-specific CTLs and m8Δ expressing SCT against CTL-resistant HTLV-I-infected cells. Our results suggested the possible application of the combined use of oncolytic viruses presenting tumor antigens and tumor-specific CTLs for the treatments against tumors including ATL.

## 2. Materials and Methods

### 2.1. Cells and Viruses

An HTLV-I-immortalized cell line, FPM1, was previously established by cocultivating thymocytes of an F344/N Jcl-rnu/+ rat (Clea Japan, Inc. Tokyo, Japan) with HTLV-I producing human cell line, MT-2, which was treated with mitomycin C (50 *μ*g/mL) for 30 min at 37°C [30]. FPM1V.EFGFP, FPM1V.EFGFP/8R, and 4O1/C8 cells were established as previously described [25]. FPM1V.EFGFP was a subclone of FPM1 cells, which stably expresses EGFP. FPM1V.EFGFP/8R cells were generated from FPM1V.EFGFP cells, by continuously cultivating with a Tax-specific CTL, 4O1/C8, and obtained an ability to evade from CTL killing by 4O1/C8 cells. FPM1 and its subclones were maintained in RPMI 1640 with 10% heat-inactivated FCS (Sigma-Aldrich, St. Louis, MO), 55 *μ*M of 2-mercaptoethanol (Gibco, Grand Island, NY), penicillin, and streptomycin. The 4O1/C8 cells were established from an F344/N Jcl-rnu/+ rat inoculated with Tax-coding DNA and were maintained in RPMI 1640 medium with 10% FCS, 55 *μ*M of 2-mercaptoethanol, and 20 U of IL-2 (PEPROTECH, London, UK) per mL with periodical stimulation using formalin-fixed FPM1 cells every 4 weeks. A rabbit kidney epithelial cell line, RK13, was cultured in RPMI1640 supplemented with 10% FCS. Hamster BHK cells were cultured in D-MEM supplemented with 10% FCS. Canarypox virus (a kind gift of National Institute of Animal Health) [31], mO, m8Δ [22], and LC16 m8ΔVNC110 that harbors multiple cloning site in the HA gene of m8Δ genome were described previously [32]. Viral titers were calculated on the basis of the number of plaques on RK13 cells.

### 2.2. Construction of m8Δ Expressing SCTs of Rat MHC-I

The expression vectors, pEF/RT1AlSCTax180L and pEF/RT1AlSCNLEnv371L, which encode SCTs of rat MHC-I with Tax180–188 (Tax180) or human immunodeficiency virus type 1 (HIV-1) NL43 Env371–379 (NLEnv371) epitopes, respectively, were previously constructed [29]. To generate m8Δ expressing SCTs, peptide-*β*_2_m-RT1A^l^ fusion sequence in pEF/RT1AlSCTax180L or pEF/RT1AlSCNLEnv371L was amplified by PCR to add CpoI and FseI sites at the 5′ and 3′ end of the fusion constructs, respectively, and were ligated into the LC16m8ΔVNC110 genome that had been digested with CpoI and FseI. The ligated DNA was transfected into BHK cells that were infected with canarypox virus, as described previously [32]. The recombinants were selected by plaque ELISA using an anti-rat MHC-I antibody (clone OX-18; BD PharMingen Co., San Diego, CA) and were then subjected to Western blotting to confirm the proper protein expression.

### 2.3. Protein Analysis

For plaque ELISA, recombinant VVs were infected to RK13 cells on 6 well plates at approximately 100 pfu/well. After incubation for 72 h at 33°C, the infected cells were fixed with 2% paraformaldehyde solution followed by permeabilization with 0.5% NP40 for 1 min. The fixed cells were blocked with 5% skim milk in PBS for 30 min and incubated with an anti-rat MHC-I antibody (clone OX-18; BD PharMingen Co.) followed by incubation with an alkaline phosphatase-conjugated anti-mouse IgG antibody (Sigma-Aldrich). After staining with alkaline phosphatase substrates, the plaques with dark blue color were collected as positive clones.

For Western blotting, cells were resuspended in ice-cold extraction buffer (20 mM HEPES [pH 7.9], 10 mM KCl, 1 mM MgCl_2_, 150 mM NaCl, 1% Triton X-100, 0.5 mM DTT, 0.5 mM PMSF, 1 *μ*g/mL aprotinin, and 1 *μ*g/mL leupeptin) and gently rocked for 30 minutes. After centrifugation at 14,000 ×g for 20 minutes at 4°C, the supernatant was collected as a whole cell extract. The protein concentration of each sample was determined using a BCA protein assay reagent kit (Pierce Biotechnology, Rockford, IL). Fifty *μ*g of whole cell extracts was separated by 8% SDS-PAGE and transferred to a nitrocellulose filter. The filter was incubated with an anti-rat MHC-I antibody and then with an anti-mouse Ig antibody conjugated to horseradish peroxidase (Amersham, Arlington Heights, IL). Antibodies bound to the filter were detected by the enhanced chemiluminescence method (Amersham).

### 2.4. A Flow-Cytometric CTL Killing Assay

EGFP-expressing target cells (2.5–5.0 × 10^4^ cells/well) were cocultured with CTLs (2.5 × 10^5^–1 × 10^6^ cells/well). These mixed cultures were immediately subjected to flow-cytometric analysis or were incubated for indicated days and then subjected to flow-cytometric analysis. Cytofluorometry was done on a FACSCalibur (BD Biosciences, San Jose, CA) and analyzed with Cell Quest software. Target cells were clearly gated away from CTLs by light-scatter properties and EGFP expression.

### 2.5. IFN-*γ* Production Assay

The 4O1/C8 (1 × 10^5^/well) was mixed with various stimulator cells (2 × 10^4^/well). After indicated period of mixed culture, supernatants were harvested and subjected to rat IFN-*γ* ELISA (eBioscience Inc., San Diego, CA) in accordance with the manufacturer's instructions.

### 2.6. Cell Viability Assay

Cells were infected with VVs and then incubated for indicated periods. In some experiments, cells were stimulated with formalin-fixed FPM1 cells for 2 days and then infected with VVs. The number of growing cells was determined by using a cell counting kit-8 (Dojinndo Laboratories, Kumamoto, Japan) in accordance with the manufacturer's instructions. Cell viabilities are expressed as percentages of cell survival of mock-infected cultures, as described previously [24].

### 2.7. Statistical Analysis

Comparisons between individual data points were made using a Student's* t*-test. Two-sided* P* values <0.05 were considered statistically significant.

## 3. Results

### 3.1. Rat HTLV-I-Infected Cells Were Susceptible to the Killing by Attenuated Vaccinia Strain, m8Δ

**Figure 1:**
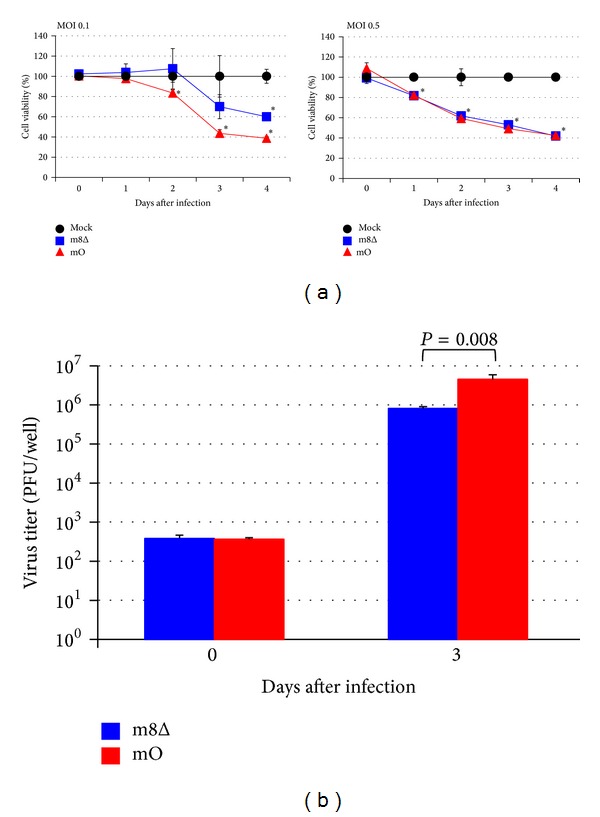
Viability of FPM1 cells infected with attenuated VVs. (a) FPM1 cells were exposed to m8Δ (■), mO (▲), or PBS (●) at indicated MOI for 2 hrs. After extensive wash, the cells were cultured for indicated periods and the cell growth was assessed by using cell counting kit 8. The cell viabilities are expressed as percentages of the cell survival of mock-infected cultures. The data are presented as mean ± SD of triplicate wells. Asterisks indicate statistical significance (*P* < 0.05) compared to the mock-infected controls. (b) The proliferation of VVs in FPM1 cells infected with the virus at MOI 0.1 was determined by titrating the cell lysates collected at indicated days. The data are presented as mean ± SD of triplicate wells. Statistical significance was determined as *P* < 0.05. Similar results were obtained in two independent experiments.

To develop a safe and effective smallpox vaccine and vector virus, we have previously constructed genetically stable m8Δ, which is less pathogenic than its parental mO due to the deletion of B5R gene [22], and successfully applied it for animal studies of HIV-1 vaccine developments [32–34]. In this study, to determine whether m8Δ possesses cytolytic activity against HTLV-I-infected cells, a rat HTLV-I-infected cell line, FPM1 was infected with m8Δ or mO. As shown in Figure 1(a), we observed the gradual reduction of cell viability in FPM1 cells infected with m8Δ at multiplicity of infection (MOI) 0.1 and confirmed the significant difference in cell viability between m8Δ - and mock-infected cells at 4 days after infection, indicating the induction of cytolysis of FPM1 cells by m8Δ. The cytolysis induction by mO was more efficient than that by m8Δ at MOI 0.1, because significant difference in cell viability was observed after 2 days of infection. Similar levels of significant cytolysis induction were observed in FPM1 cells infected with either m8Δ or mO at MOI 0.5. We next examined the virus replication in the cells infected with VV at MOI 0.1 and confirmed 2.1 × 10^3^ and 1.2 × 10^4^ fold increase of infectious m8Δ and mO, respectively, at 3 days after infection (Figure 1(b)). These results indicate that the attenuated m8Δ possesses lower level of oncolytic activity compared with its parental mO and that increasing the virus inoculum can compensate the reduced activity. In addition, we have maintained the virus-infected cells for extended periods and confirmed that all the cells used in Figure 1(a) were eventually killed by VVs (data not shown). Thus, prolonged cultivation could also improve the efficacy of oncolysis by highly attenuated VVs.

### 3.2. A Tax-Specific CTL Line, 4O1/C8, Was Resistant to Killing by m8Δ

**Figure 2:**
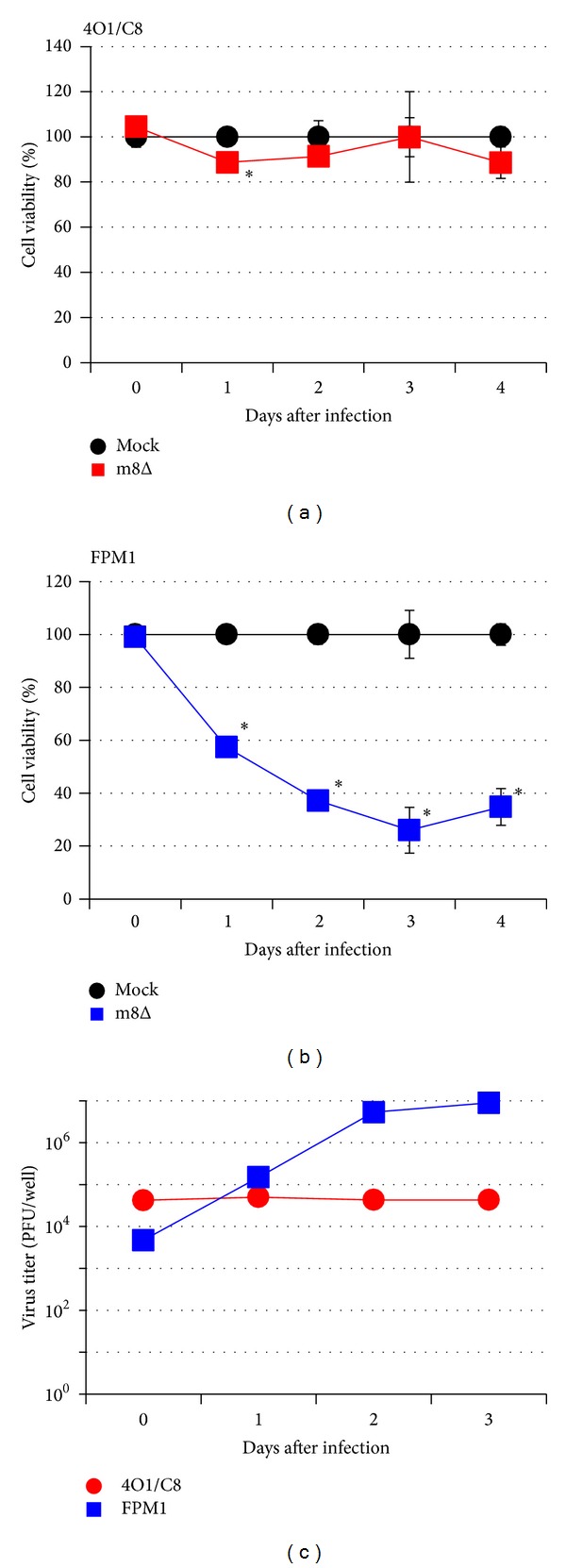
A Tax-specific CTL line, 4O1/C8, was resistant to killing by m8Δ. The 4O1/C8 (a) or FPM1 (b) cells were exposed to m8Δ (■) or PBS (●) at MOI 2 for 2 hrs. After extensive wash, the cells were cultured for indicated periods and the cell growth was assessed by using cell counting kit 8. The cell viabilities are expressed as percentages of the cell survival of mock-infected cultures. The data are presented as mean ± SD of triplicate wells. Asterisks indicate statistical significance (*P* < 0.05) compared to the mock-infected controls. Similar results were obtained in two independent experiments. (c) The proliferation of VVs in 4O1/C8 (●) or FPM1 (■) cells infected with the virus at MOI 2 was determined by titrating the cell lysates collected at indicated days. The data are presented as mean of duplicate wells.

Virus-specific CTLs that play important roles in eradication of virus-infected cells should not be eliminated by oncolytic viruses during treatment. Thus, it is important to confirm the resistance of CTLs to cytolysis by m8Δ. To assess the susceptibility of Tax-specific CTLs to killing by m8Δ, 4O1/C8 cells were stimulated with formalin-fixed FPM1 cells to induce cell proliferation and then were exposed to m8Δ at MOI 2. As shown in Figure 2(a), exposure of 4O1/C8 to m8Δ did not influence the growth of the CTLs. In contrast, dramatic decrease in the viability of FPM1 was observed after infection of m8Δ at MOI 2 (Figure 2(b)). The enhanced cytolysis of FPM1 should be due to higher amount of inoculated virus compared with that used in Figure 1. The assessment of virus titer in the virus-exposed 4O1/C8 demonstrated that the titer of 4O1/C8-associated virus was stable during the first 4 days, suggesting that m8Δ was not able to proliferate in the CTLs but was stable for several days in the presence of the CTLs (Figure 2(c)). Alternatively, it is also possible that low levels of m8Δ proliferation may compensate the natural reduction of the virus titer in the culture. These results indicated that 4O1/C8 is resistant to the cytolysis by m8Δ and suggested that virotherapy using m8Δ does not affect the function of CTLs. Thus, m8Δ could be applicable for the combination therapies using oncolytic viruses and antigen-specific T cells against HTLV-I-infected cells.

### 3.3. Lack of IFN-*γ* Production Was Correlated with the Resistance of FPM1V.EFGFP/8R Cells to Killing by 4O1/C8 CTL

**Figure 3:**
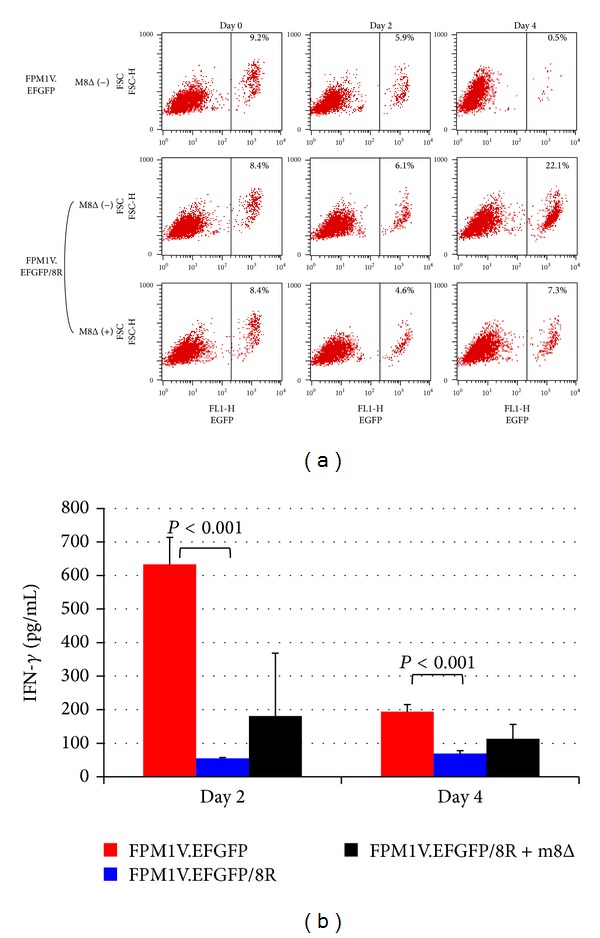
Lack of IFN-*γ* production was correlated with the resistance of FPM1V.EFGFP/8R cells to killing by 4O1/C8. (a) FPM1V.EFGFP or FPM1V.EFGFP/8R cells (5 × 10^4^/well) were mixed with 4O1/C8 cells (5 × 10^5^/well) at an E : T ratio of 10 : 1 in the presence or absence of m8Δ (1 × 10^5^ PFU/well) and subjected to flow-cytometric analysis for the expression of EGFP at the indicated days. Percentage of EGFP positive cells is indicated in each panel. (b) Production of IFN-*γ* in the supernatants of mixed culture prepared in (a) was measured by ELISA at the indicated days of culture. The data represent the mean ± SD of triplicate wells. Statistical significance was determined as *P* < 0.001. Similar results were obtained in two independent experiments.

We have previously established an assay system by which we can evaluate the susceptibility of HTLV-I-infected cells to CTL killing by flow-cytometric analysis [25]. In this study, we used EGFP-expressing subclones of FPM1 cells, FPM1V.EFGFP and FPM1V.EFGFP/8R, as target cells of Tax-specific CTLs. FPM1V.EFGFP/8R cells were previously isolated by continuously cocultivating with 4O1/C8 cells and were shown to be resistant to killing by 4O1/C8 due to downregulation of MHC-I but not Tax expression [25]. As we have previously reported, mixed culture of FPM1V.EFGFP and 4O1/C8 cells resulted in the dramatic decrease of EGFP-positive FPM1V.EFGFP fractions (Figure 3(a)). In contrast, the percentage of FPM1V.EFGFP/8R increased in 4 days of mixed culture with 4O1/C8 cells, indicating the resistance of FPM1V.EFGFP/8R to killing by 4O1/C8. To determine whether the activation of 4O1/C8 was induced in the mixed culture, IFN-*γ* production in the supernatants was evaluated. As shown in Figure 3(b), mixed culture of 4O1/C8 cells with FPM1V.EFGFP induced IFN-*γ* secretion whereas that with FPM1V.EFGFP/8R did not. Thus, production of IFN-*γ* in the mixed culture correlated with killing of the HTLV-I-infected cells by 4O1/C8. We next infected the mixed culture of 4O1/C8 and FPM1V.EFGFP/8R cells with m8Δ to determine whether cytolysis of the CTL-resistant cells by the oncolytic virus induced the activation of the Tax-specific CTLs. As shown in Figure 3(a), slight decrease of GFP positive cell fraction (7.4 ± 0.2%) was observed in 4 days of mixed culture with m8Δ, which was in stark contrast to the apparent increase of GFP positive cell fraction in the absence of m8Δ  (22.4 ± 0.3%), demonstrating that cytolysis of FPM1V.EFGFP/8R was induced by m8Δ. There were no cells surviving after extended cultivation of the virus-infected FPM1V.EFGFP/8R cells in the experiment (data not shown). However, IFN-*γ* production was not detected in the mixed culture with m8Δ (Figure 3(b)), indicating that cytolysis of the HTLV-I-infected cells by m8Δ was independent of CTL activation.

### 3.4. Characterization of Recombinant VVs Expressing SCTs of Rat MHC-I

**Figure 4:**
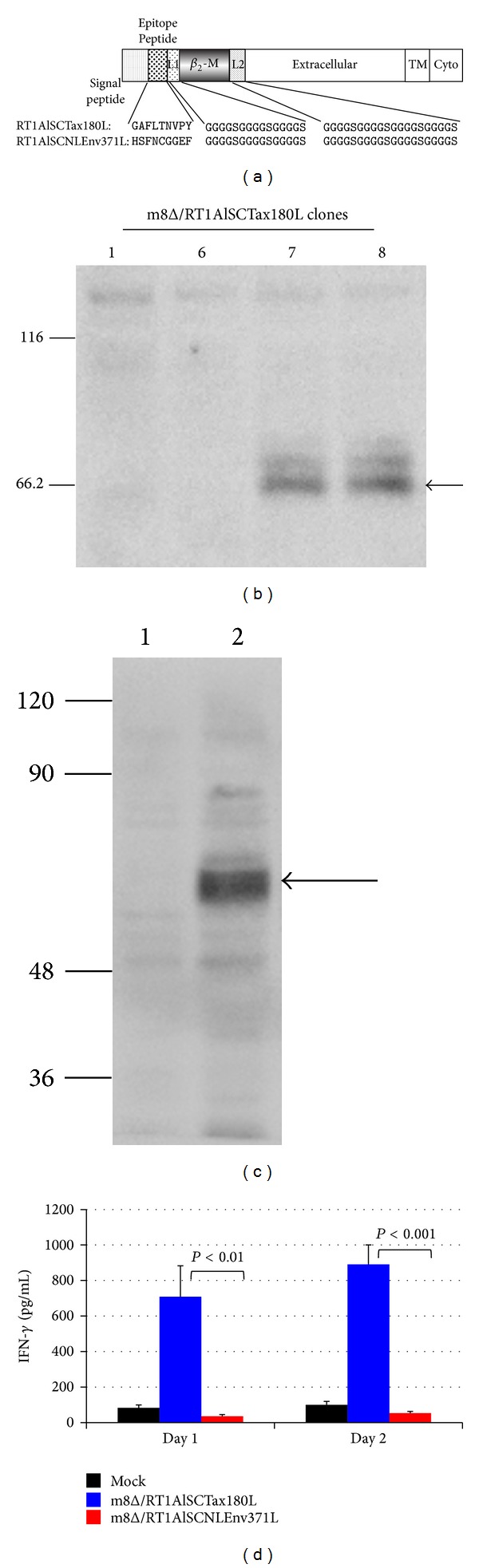
Characterization of m8Δ expressing SCTs. (a) Diagram of SCTs encoding Tax180–188 or NLEnv371–379 linked to *β*_2_m and RT1.A^l^ molecules with two linkers. L1, linker 1; L2, linker 2; TM, transmembrane region; Cyto, cytoplasmic region. ((b) and (c)) Whole cell extracts were isolated from RK13 cells infected with indicated clones of m8Δ/RT1AlSCTax180L (b) or RK13 cells infected with m8Δ (lane 1) or m8Δ/RT1AlSCNLEnv371L (lane 2) (c) and 50 *μ*g of each lysate was subjected to Western blotting analysis for the expression of the SCT of MHC-I proteins. Arrows indicate the SCT of MHC-I proteins detected by an anti-rat MHC-I antibody. Molecular weight markers are indicated (kDa) on the left margin. (d) RK13 cells were exposed to m8Δ/RT1AlSCTax180L, m8Δ/RT1AlSCNLEnv371L, or PBS for 2 hrs. After extensive wash, the cells were cocultivated with 4O1/C8 for indicated days. Production of IFN-*γ* in the supernatants was measured by ELISA. The data represent the mean ± the SD of triplicate wells. Statistical significance was determined as *P* < 0.01. Similar results were obtained in two independent experiments.

To improve the efficiency of oncolytic viruses, various types of modifications have been reported [17]. In this study, we have utilized SCTs with a Tax-epitope to enhance the oncolytic ability of m8Δ against HTLV-I-infected cells in combination with Tax-specific CTLs. Tax180 epitope was previously identified as an RT1.A^l^-restricted CTL epitope recognized by a Tax-specific CTL line, 4O1/C8 [14]. As a negative control, we have chosen a putative RT1.A^l^-restricted epitope in the envelope of HIV-1 NL4-3 strain, NLEnv371, which was determined to have the same point as Tax180 epitope scored by epitope prediction data via http://www.syfpeithi.de/ [35]. A schematic representation of SCTs is shown in Figure 4(a). We have introduced the coding sequence of SCTs with Tax180 or NLEnv371 into the genome of m8Δ and obtained m8Δ/RT1AlSCTax180L or m8Δ/RT1AlSCNLEnv371L, respectively. The SCT protein expression by m8Δ/RT1AlSCTax180L was examined in RK13 cells. Among the 4 clones tested, 2 clones (Numbers 7 and 8) appeared to express SCTs and clone Number 7 was used for further studies (Figure 4(b)). The expression of SCTs by m8Δ/RT1AlSCNLEnv371L was also confirmed by Western blotting (Figure 4(c)). We further assessed the function of the SCTs expressed by m8Δ/RT1AlSCTax180L, by infecting the virus to RK13 cells and coculturing the infected cells with 4O1/C8. As shown in Figure 4(d), RK13 cells infected with m8Δ/RT1AlSCTax180L were able to induce IFN-*γ* secretion by 4O1/C8. In contrast, RK13 cells infected with m8Δ/RT1AlSCNLEnv371L induced little amount of IFN-*γ* secretion by the Tax-specific CTLs. These results indicated that SCTs expressed by m8Δ/RT1AlSCTax180L were able to activate Tax-specific CTLs. Thus, it is expected that m8Δ/RT1AlSCTax180L possesses dual functions of both lysing HTLV-I-infected cells and activating Tax-specific CTLs.

### 3.5. Combined Effects of 4O1/C8 and m8Δ on Killing of CTL-Resistant HTLV-I-Infected Cells

**Figure 5:**
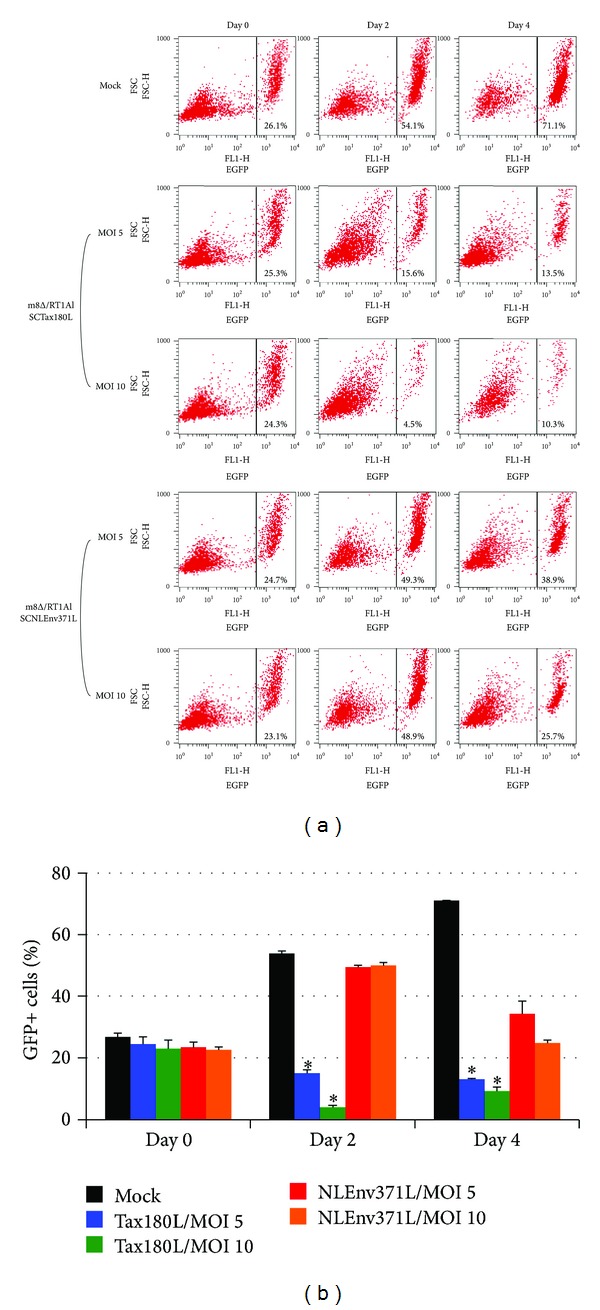
Combined effects of 4O1/C8 and m8Δ on killing of CTL-resistant HTLV-I-infected cells. (a) FPM1V.EFGFP/8R cells were exposed to m8Δ/RT1AlSCTax180L, m8Δ/RT1AlSCNLEnv371L, or PBS for 2 hrs at indicated MOI. After extensive wash, the cells were cocultivated with 4O1/C8 for indicated periods and subjected to flow-cytometric analysis for the expression of EGFP at the indicated days. Percentage of EGFP positive cells is indicated in each panel. (b) Bar graph of the flow-cytometric data. The data are presented as mean ± SD of triplicate wells. Asterisks indicate statistical significance (*P* < 0.01) compared to the m8Δ/RT1AlSCNLEnv371L-infected cells with corresponding MOI.

**Figure 6:**
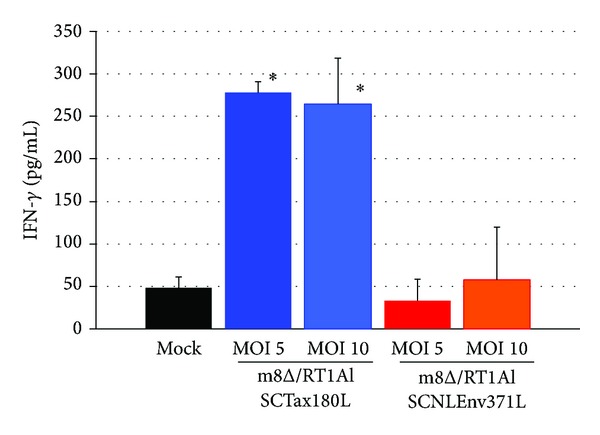
Production of IFN-*γ* by 4O1/C8 that was cocultured with recombinant m8Δ-exposed FPM1V.EFGFP/8R cells. FPM1V.EFGFP/8R cells were exposed to m8Δ/RT1AlSCTax180L, m8Δ/RT1AlSCNLEnv371L, or PBS for 2 hrs at indicated MOI. After extensive wash, the cells were cocultivated with 4O1/C8 for 2 days. Production of IFN-*γ* in the supernatants of mixed culture was measured by ELISA. The data represent the mean ± SD of triplicate wells. Asterisks indicate statistical significance (*P* < 0.05) compared to the mock-infected controls.

**Figure 7:**
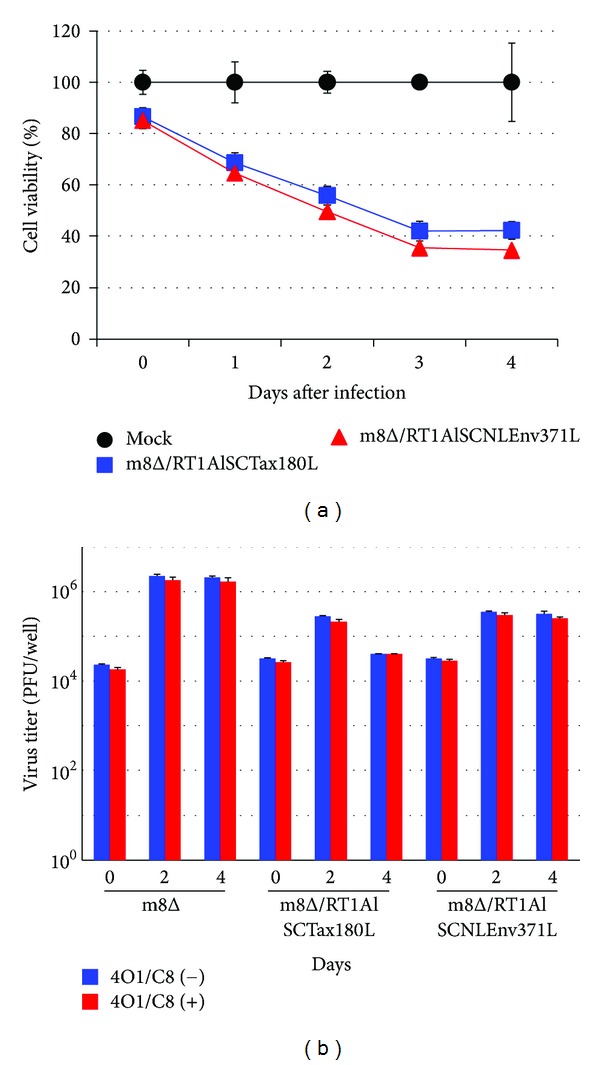
Viability of and viral replication in FPM1V.EFGFP/8R cells exposed to recombinant m8Δs. (a) FPM1V.EFGFP/8R cells were exposed to m8Δ/RT1AlSCTax180L (■), m8Δ/RT1AlSCNLEnv371L (▲), or PBS (●) for 2 hrs at MOI 5. After extensive wash, the cells were cultured for indicated periods and the cell growth was assessed by using cell counting kit 8. The cell viabilities are expressed as percentages of the cell survival of mock-infected cultures. The data are presented as mean ± SD of triplicate wells. (b) FPM1V.EFGFP/8R cells (5 × 10^4^/well) were exposed to m8Δ at MOI 2, or m8Δ/RT1AlSCTax180L, or m8Δ/RT1AlSCNLEnv371L at MOI 5. After extensive wash, the cells were cultured in the presence or absence of 4O1C8 cells (2.5 × 10^5^/well) for indicated periods and were collected for the evaluation of virus proliferation. The proliferation of VVs was determined by titrating the cell lysates. The data are presented as mean ± SD of triplicate wells.

To examine the combined effects of Tax-specific CTLs and m8Δ expressing SCTs, we next infected FPM1V.EFGFP/8R cells with m8Δ/RT1AlSCTax180L or m8Δ/RT1AlSCNLEnv371L and cocultivated the infected cells with 4O1/C8. As shown in Figure 5, the proportion of FPM1V.EFGFP/8R cells in the mixed culture clearly decreased at 4 days after m8Δ/RT1AlSCNLEnv371L infection compared to the mock-infected controls. The decrease of FPM1V.EFGFP/8R cells was MOI-dependent and may be due to oncolytic ability of the virus, since IFN-*γ* production was not detected in the mixed culture (Figure 6). A greater reduction of EGFP-positive cells was observed in the mixed culture of FPM1V.EFGFP/8R cells infected with m8Δ/RT1AlSCTax180L. In particular, m8Δ/RT1AlSCTax180L infection at MOI 10 induced most dramatic elimination of FPM1V.EFGFP/8R cells. This may be due to the combined effects of oncolytic activity and activation of 4O1/C8 cells induced by SCTs with Tax presentation, since IFN-*γ* production was clearly induced in the mixed culture (Figure 6). Induction of IFN-*γ* was partly due to the direct effect of m8Δ/RT1AlSCTax180L to 4O1/C8, since direct exposure of 4O1/C8 to m8Δ/RT1AlSCTax180L, but not to m8Δ/RT1AlSCNLEnv371L, resulted in the production of IFN-*γ* (data not shown). We also examined the cytolytic activity of m8Δs expressing SCTs in the absence of 4O1/C8 to determine whether expression of different epitopes affects the lysis of FPM1V.EFGFP/8R cells. As shown in Figure 7(a), equivalent levels of cell growth inhibition were observed in FPM1V.EFGFP/8R cells infected with either m8Δ/RT1AlSCTax180L or m8Δ/RT1AlSCNLEnv371L. These results demonstrated that there is no difference in direct cytolytic ability between m8Δ/RT1AlSCTax180L and m8Δ/RT1AlSCNLEnv371L and further indicated that significantly strong reduction of EGFP-positive cell fraction observed in the mixed culture of 4O1/C8 and m8Δ/RT1AlSCTax180L-infected FPM1V.EFGFP/8R cells was due to the additional cytotoxic activity of 4O1/C8 activated by m8Δ/RT1AlSCTax180L-mediated epitope presentation. Finally, we have evaluated the virus titers in FPM1V.EFGFP/8R cells infected with VVs in the presence or absence of 4O1/C8 cells to determine whether the CTLs influence the replication of VVs. As shown in Figure 7(b), we have not observed any significant differences of the virus titer between VV-infected FPM1V.EFGFP/8R cells cultivated with 4O1/C8 and those without 4O1/C8 during the first 4 days after infection regardless of the VVs used, although slight reduction of VV titer was induced by the addition of 4O1/C8 cells in most of the samples examined. Thus, the CTLs did not significantly affect the replication of VVs in the present experiments.

## 4. Discussion

The primary effect of oncolytic virotherapy depends on the vigorous viral replication and spread within tumor tissues. In addition, it has been reported that oncolytic virus-mediated tumor destruction leads to the activation of tumor-specific immune responses and the improved efficacy of virotherapy [36, 37]. Thus, activation of tumor-specific immune responses could be another strategy to enhance tumor specific killing by attenuated oncolytic viruses. Indeed, GM-CSF-encoding VV or herpes virus has been developed to effectively induce tumor regression [38, 39]. Encoding a tumor antigen within an oncolytic virus also enhanced the tumor-specific immune responses and the efficacy of tumor eradication [40, 41]. Based on these previous reports, we have developed a novel combination therapy against HTLV-I tumor in a rat model system, which consists of a Tax-specific CTL line and an attenuated VV expressing SCTs with a Tax-epitope. In line with the previous reports, our present results demonstrated the effective cytolysis of HTLV-I-infected cells by an attenuated VV and the synergistic effects between activated virus-specific T cells and oncolytic viruses toward eliminating tumor cells. Introduction of SCT system should be extremely important in the case of HTLV-I infection, because repression of Tax expression is frequently observed in HTLV-I carriers and is a possible cause of the declined virus-specific immune responses [8]. By using SCTs, effective presentation of Tax-epitopes could be expected to induce proper activation of anti-HTLV-I T cell responses, even when Tax expression is repressed. Since repression of antigen presentation by MHC-I represents a common feature of tumor cells and is one of the mechanisms of tumor immune evasion, encoding a SCT of MHC-I gene within an attenuated oncolytic virus could also be effective against a broad range of tumors. Moreover, it has been reported that combining adoptive cell therapy with oncolytic viruses leads to effective elimination of tumor cells [42]. In addition to the primary effects of direct killing of tumor cells, tumor-specific T cells were also known to enhance oncolysis in vivo by carrying oncolytic viruses to distal tumor sites [43]. Thus, combined administration of SCT-expressing oncolytic virus and tumor-specific T cells could be one of the ideal combinations to maximize antitumor effects. However, it is also important to note that CTLs may possibly have inhibitory effects on the replication of oncolytic viruses. Although significant inhibition of the virus replication was not induced by 4O1/C8 cells as shown in Figure 7(b), IFN-*γ* produced by the CTLs could have the potential to exert adverse effects on the virus replication in different conditions. Thus, it should be important to consider the effects of CTLs on virus replication and oncolytic activity when we design the combination therapy of antigen-expressing oncolytic viruses and tumor-specific T cells.

VVs are known to have unique biological properties, including resistance to antibody- and compliment-mediated neutralization abilities in blood [44, 45]. Taking advantage of the properties, a recent clinical trial has successfully demonstrated the intravenous delivery and replication of oncolytic VVs in metastasized tumor tissue [21]. Since HTLV-I primarily infects T cells in the blood and expands through the bloodstream, it is reasonable to assume that VVs could also reach systemically spreading HTLV-I-infected cells after intravenous administration. In addition, the unique biological nature of VVs made them possible to spread systemically even in patients with a history of live VV vaccination [21]. Thus, it is anticipated that oncolytic VVs could be effective in HTLV-I-infected individuals with anti-VV immunity.

The efficacy of a cancer treatment has to be balanced against its potential toxicity to normal cells. The safety of m8Δ has been demonstrated through the use of its natural counterpart, m8, for smallpox vaccine [23]. It is of note that m8Δ infection showed neither viral replication in nor cytolysis of Tax-specific CTLs (Figure 2), further indicating the reduced toxicity to normal cells. However, due to the lack of* B5R* gene, oncolytic activity of m8Δ was shown to be declined [24]. Our present observation also confirmed the weaker oncolytic activity of m8Δ than that of mO in a rat HTLV-I-infected cell line (Figure 1). In addition, we observed the lack of Tax-specific T cell activation in the mixed culture of FPM1V.EFGFP/8R cells infected with m8Δ (Figure 3) or m8Δ/RT1AlSCNLEnv371L (Figure 6). Since FPM1V.EFGFP/8R cells are known to express Tax protein [25], it is anticipated that cytolysis of FPM1V.EFGFP/8R by m8Δ should lead to the release of the viral antigen and the activation of 4O1/C8 cells. Indeed, our previous results showed that addition of Tax180 peptide directly to the culture led to the activation of 4O1/C8 [25]. Thus, it is possible that cytolysis of FPM1V.EFGFP/8R cells by m8Δ was slowly processed even when m8Δ efficiently replicated in the cells and that the amount of Tax protein released from destructed cells was not enough to activate Tax-specific CTLs in the present experimental condition. Under this condition, to compensate the reduced oncolytic ability of m8Δ, we introduced a gene encoding SCT with Tax180 within the genome of m8Δ and showed the improved cytolysis of FPM1 cells by Tax-specific CTLs without altering direct oncolytic ability of m8Δ. It seemed that the ability to induce T cell activation by m8Δ/RT1AlSCTax180L could be fairly strong, because rabbit RK13 cells also became capable of inducing IFN-*γ* production by 4O1/C8 cells after m8Δ/RT1AlSCTax180L infection (Figure 4(d)). Since uncontrolled activation of T cells may result in normal cell injury, it is also necessary to carefully evaluate the safety of the virus in vivo. In addition, there are other strategies to overcome the reduced oncolytic activity of attenuated viruses [17]. As for m8Δ, Hikichi et al. have successfully developed a microRNA-regulated system, by which m8Δ can selectively express B5R in tumor cells and demonstrated full restoration of its oncolytic activity [24]. These strategies could be also combined to further enhance oncolytic activities without damaging normal cells.

ATL is known to acquire resistance to conventional chemotherapy and has a poor prognosis. Although allo-HSCT had been developed for the treatment of ATL, patients who are eligible for the treatment are still limited [46, 47]. Recently, a novel promising therapy using a humanized anti-CCR4 monoclonal antibody has been reported to be effective against ATL [48]. In addition, there are other novel target proteins discovered for T cell therapies against ATL, including NY-ESO-1 [49], and hTERT [50]. Since the VV encoding SCTs developed in this study as well as previously reported VSV [19] have unique mechanisms of action against HTLV-I-infected cells, it is possible that combination of these oncolytic viruses with the recently developed immune therapies could further enhance the efficacy of ATL treatment.

## 5. Conclusion

We demonstrated that an attenuated VV, m8Δ, possesses oncolytic activity to HTLV-I-infected cells and that m8Δ encoding SCT with a Tax-epitope enhances the cytolysis of CTL-resistant HTLV-I-infected cells in combination with Tax-specific CTLs. This newly established VV expressing SCT should have combining tumor debulking activity of direct tumor lysis and activation of tumor specific CTLs. The combination of epitope specific-CTLs and attenuated VVs encoding SCTs with corresponding epitopes could be effective tool to eradicate tumors escaping from immune surveillance.

## Abbreviations

ATL: Adult T cell leukemia
allo-HSCT: Allogeneic hematopoietic stem cell transplantation
*β*_2_m: *β*_2_-microgrobulin
CTL: Cytotoxic T lymphocyte
HIV-1: Human immunodeficiency virus type 1
HTLV-I: Human T cell leukemia virus type I
HAM/TSP: HTLV-I-associated myelopathy/tropical spastic paraparesis
MOI: Multiplicity of infection
MHC-I: Major histocompatibility complex class I
SCT: Single chain trimer
VV: Vaccinia virus
VSV: Vesicular stomatitis virus.
